# Hernandezine induces autophagic cell death in human pancreatic cancer cells via activation of the ROS/AMPK signaling pathway

**DOI:** 10.1038/s41401-022-01006-1

**Published:** 2022-10-25

**Authors:** Chang-feng Song, Yu-heng Hu, Zhi-guo Mang, Zeng Ye, Hai-di Chen, De-sheng Jing, Gui-xiong Fan, Shun-rong Ji, Xian-jun Yu, Xiao-wu Xu, Yi Qin

**Affiliations:** 1 Department of Pancreatic Surgery, Fudan University Shanghai Cancer Center, Shanghai, 200032 China; 2 Department of Oncology, Shanghai Medical College, Fudan University, Shanghai, 200032 China; 3 Shanghai Pancreatic Cancer Institute, Shanghai, 200032 China; 4 Pancreatic Cancer Institute, Fudan University, Shanghai, 200032 China; 5 Yujie Biotechnology (Shanghai) Co., Ltd., Shanghai, 201314 China

**Keywords:** pancreatic cancer, hernandezine, ROS, autophagy, autophagic cell death

## Abstract

Hernandezine (Her) is a bisbenzylisoquinoline alkaloid extracted from the traditional Chinese herbal medicine *Thalictrum glandulosissimum*. Evidence shows that Her is a natural agonist of adenosine monophosphate (AMP)-activated protein kinase (AMPK) and induces apoptosis and autophagy in tumor cells. In this study, we investigated the role of autophagy in Her-induced cell death in human pancreatic cancer cell lines. We showed that Her dose-dependently suppressed cell proliferation, promoted autophagy and induced autophagic death in pancreatic ductal adenocarcinoma (PDAC) cell lines Capan-1 and SW1990. The IC_50_ values of Her in inhibition of Capan-1 and SW1990 cells were 47.7 μM and 40.1 μM, respectively. Immunoblotting showed that Her (1−40 μM) promoted the conversion of LC3-I to LC3-II, and Her exerted concentration-dependent and time-dependent effects on autophagy activation in PDAC cells. In transmission electron microscopy and fluorescence image analysis, we found that autophagic vacuoles were significantly increased in Her-treated cells. Knockdown of ATG5, a key gene in the autophagy pathway, alleviated the activation of autophagy by Her. These results demonstrated that Her induced autophagy in PDAC cells. Intensely activated autophagy could promote cell death. The autophagy inhibitors, BafA1 and HCQ significantly inhibited Her-induced cell death, implying that Her induced autophagic cell death in PDAC cells. Moreover, we showed that Her activated autophagy by increasing the phosphorylation of AMPK and decreasing the phosphorylation of mTOR/p70S6K. Knockdown of AMPKα relieves the autophagic cell death induced by Her. Furthermore, Her concentration-dependently enhanced reactive oxygen species (ROS) generation in PDAC cells. Antioxidants could reduce the phosphorylation of AMPK and suppress autophagic cell death induced by Her. Our study provides evidence for the development of Her as a therapeutic agent for the treatment of pancreatic cancer.

## Introduction

Pancreatic cancer is a tumor with relatively high malignancy and mortality rates [1]. Pancreatic ductal adenocarcinoma (PDAC) accounts for more than 90% of pancreatic cancer cases. The number of patients diagnosed with pancreatic cancer in China is increasing year by year, but only approximately 20% of patients have access to surgery. In recent years, although research is ongoing and the clinical diagnosis and treatment of pancreatic cancer have improved, the 5-year survival rate of pancreatic cancer patients has not significantly improved [2]. Few chemotherapeutic options are available for pancreatic cancer treatment [3]. Therefore, the development and identification of effective drugs for the treatment of pancreatic cancer is of clinical significance.

Hernandezine (Her), a bisbenzylisoquinoline alkaloid, was isolated from the herbal plant *Thalictrum glandulosissimum* [4]. Pharmacological studies have shown that Her has a variety of biological activities [5–9], but its extraction process is complicated and its natural origin is limited, so Her has not been developed into a clinical medicine for treatment. Her has antitumor [10], antiplatelet aggregation [11] and calcium channel blocking effects [12, 13], but the mechanism of its action has not been fully elucidated. Law et al. reported that Her is an adenosine monophosphate-activated protein kinase (AMPK) agonist that induces apoptosis and autophagy and promotes tumor cell death [10]. Other reports showed that Her reverses the multidrug resistance of tumor cells as an effective multidrug resistance reversal agent [14].

Autophagy is a process in which cells remove damaged proteins and organelles and recycle intracellular substances and energy. A normal level of autophagy is required for cells to maintain a stable intracellular environment [15, 16]. However, autophagy is activated in lesions, diseases and tumors in response to stresses such as nutrient shortages, infections, and specific signaling pathways [17, 18]. There are various types of autophagy, including macroautophagy, microautophagy and molecular chaperone-mediated autophagy. Macroautophagy is characterized by the formation of autophagosomes with a double organelle membrane in cells; the substances to be degraded are digested within the autophagosomes [19]. Autophagosomes fuse with lysosomes and use the hydrolytic enzymes provided by lysosomes to degrade the autophagosomal contents to produce free amino acids and lipids, which are then recycled back to the cytoplasm to meet the cellular metabolic demands during nutrient deficiency [20]. Autophagy is a protective mechanism for cells, but it also plays a role in promoting cell death [21, 22].

AMPK is a sensor of cellular energy metabolism that plays an important role in the control of cellular energy homeostasis in response to external stress [23, 24]. Mammalian target of rapamycin (mTOR) is one of the downstream targets regulated by AMPK, mTOR regulates cell growth, proliferation and protein synthesis by activating downstream signaling pathways and exerts a negative regulatory effect on autophagy [25–27]. Phosphorylated mTOR inhibits the activation of autophagy, which is mainly achieved by phosphorylation of p70S6K [28]. Previous studies have found that Her induces apoptosis, causes excessive activation of autophagy, and leads to autophagic cell death in tumor cells *via* the AMPK/mTOR signaling pathway [10]. Analogs of Her have been reported to induce reactive oxygen species (ROS) production in cells [29–31]. In the autophagy induction stage, ROS activate AMPK, and activated AMPK inhibits the phosphorylation of mTOR-p70S6K, thereby inducing autophagy [32–34].

In this study, we investigated the role of autophagy in Her-induced cell death in pancreatic cancer and showed that Her promoted ROS production and induced autophagic death.

## Materials and methods

### Cell culture

The human pancreatic cancer cell lines Capan-1 and SW1990 were purchased from the American Type Culture Collection (ATCC, Manassas, VA, USA). Both cell lines were cultured in Dulbecco’s modified Eagle’s medium (Gibco, Thermo Fisher Scientific, Waltham, MA, USA) supplemented with 10% fetal bovine serum (Gibco), 100 IU/mL penicillin (Gibco) and 100 μg/mL streptomycin (Gibco). Cells were cultured at 37 °C in a humidified atmosphere of 5% CO_2_. In serum starvation experiments, cells were washed twice with phosphate-buffered saline (PBS) and once with modified Earle’s balanced salt solution (EBSS) medium (Sigma-Aldrich, St. Louis, MO, USA) and then incubated with EBSS medium at 37 °C.

### Chemicals and antibodies

The compound Her, synthesized by Dr. Zhi-guo Mang, was dissolved in dimethyl sulfoxide (DMSO) as a 50 mM stock solution and stored at −80 °C. Bafilomycin (BafA1, #HY-100558), hydroxychloroquine sulfate (HCQ, #HY-B1370) and 2′,7′-dichlorodihydrofluorescein diacetate (H_2_DCFDA, #HY-D0940) were purchased from MedChemExpress (Monmouth Junction, NJ, USA). Compound C (CC, S7306) was purchased from Selleck Chemicals (Houston, TX, USA). N-acetylcysteine (NAC, #S0077) and the Annexin V-FITC apoptosis detection kit (#C1062L) were purchased from Beyotime Biotechnology (Shanghai, China). Primary antibodies against p-AMPKα (Thr172, 1:1000, #2535) and p-p70S6K (Thr389, 1:1000, #9234) were obtained from Cell Signaling Technologies (Danvers, MA, USA). Antibodies against LC3 (1:1000, #14600-1-AP) and SQSTM1/p62 (1:1000, #18420-1-AP) were obtained from Proteintech Group (Wuhan, Hubei, China). The following antibodies were obtained from Abclonal Technology (Wuhan, China): ATG5 (:1000, #A0203), AMPKα (1:1000, #A12718), p-mTOR (Ser2448, 1:1000, #AP0094), mTOR (1:1000, #A2445), p70S6K (1:1000, #A2190), β-actin (1:5000, #AC038) and horseradish peroxidase-labeled goat anti-rabbit immunoglobulin G (IgG) (H + L) (1:5000, #AS014).

### Cell viability assay

The Cell Counting Kit-8 (CCK-8, #C0038, Beyotime) was used to determine cell viability. Capan-1 and SW1990 cells were seeded into 96-well plates (5 × 10^3^ cells per well) and cultured in 100 μL medium overnight at 37 °C. Cells were treated with various concentrations of Her (0 to 160 μM) for 24 h or 48 h. Next, the cells were incubated with 10 μL CCK-8 for another 2 h. Finally, the absorbance value of each well at 450 nm was detected using a microplate reader (SpectraMax ABS Plus, Molecular Devices, LLC., San Jose, CA, USA).

### Western blot analysis

Cells were lysed using radioimmunoprecipitation assay (RIPA) buffer (#P0013D, Beyotime), and the concentration of total protein was measured by a bicinchoninic acid protein quantitative assay kit (#P0010, Beyotime). Approximately 30 μg of proteins were subjected to sodium dodecyl sulfate polyacrylamide gel electrophoresis and then transferred onto a polyvinylidene fluoride membrane (Millipore, Billerica, MA, USA). Membranes were blocked with 5% skim milk in Tris-buffered saline containing 0.1% Tween 20 for 2 h at room temperature. The membranes were incubated with specific primary antibodies overnight at 4 °C. The membranes were then incubated with the corresponding horseradish peroxidase-labeled secondary antibody at room temperature for 2 h. Protein bands were identified using the enhanced Tanon 5200 Multi chemiluminescence (ECL) system (Tanon Science and Technology, Shanghai, China). The bands were quantified using ImageJ software (National Institutes of Health, Bethesda, MD, USA) and β-actin was used as an internal control.

### Fluorescence microscopy

The stub-RFP-sens-GFP-LC3 lentivirus was obtained from GeneChem Co. Ltd (Shanghai, China). To construct stable cell lines expressing stub-RFP-sens-GFP-LC3, Capan-1 and SW1990 cells plated in 96-well plates were cultured overnight and then infected with lentivirus. The multiplicity of infection was 20 for Capan-1 and SW1990 cells. Infected cells were selected by 3 μg/mL puromycin (Solarbio Life Science, Beijing, China). When GFP-RFP-LC3 autophagic puncta were detected, cells were cultured on 24-well cell cover glasses, fixed with 4% paraformaldehyde for 15 min, and stained with 1 μg/mL diamidino-2-phenyl indole for 10 min. The staining process was performed at room temperature and cells were protected from light. The GFP-RFP-LC3 autophagic puncta were examined using a Leica SP5 confocal microscope (Leica Microsystems Inc., Germany) with a 63× oil immersion lens.

### Transmission electron microscopy

Capan-1 and SW1990 cells were fixed with 2.5% glutaraldehyde at room temperature in the dark. The cells were gently scraped off culture dishes with a cell scraper along one direction, collected by centrifugation, resuspended in 2.5% glutaraldehyde and stored at 4 °C. The cells then underwent fixation, dehydration, infiltration embedding, polymerization, slicing and staining. The slice thickness was 60–80 nm. The sections were observed under a transmission electron microscope (TEM, HT7800, Hitachi, Japan) and images were collected for analysis.

### Autophagic cell death

Cell death was measured using an Annexin V-FITC apoptosis detection kit. Briefly, Capan-1 and SW1990 cells were digested with trypsin and resuspended in Annexin V binding buffer. The cell suspension was incubated with 5 μL of Annexin V-FITC for 15 min at room temperature and away from light. Then, 10 μL of propidium iodide solution was added to the cell suspension, and the sample was mixed gently and incubated for 5 min. The percentage of cell death was determined using flow cytometry (CytoFLEX, Beckman Coulter), and the data were analyzed with Flowjo software.

### Small interfering RNA

SiATG5 and siAMPKα were purchased from RiboBio Co., Ltd. (Guangdong, China). The target sequences were as follows: ATG-5: 5ʹ-GCTCTTCCTTGGAACATCA-3ʹ; and AMPKα: 5ʹ-GTGGAACCCTTCCATTTGA-3ʹ. Capan-1 and SW1990 cells were cultured in 12-well plates and transiently transfected with 150 nM siATG5 or siAMPKα using riboFECT™ CP Reagent (#C10511-05, RiboBio) in accordance with the manufacturer’s instructions.

### Measurement of ROS level

Capan-1 and SW1990 cells were treated with various concentrations of Her (0, 5, 10, 20, 30, 40 μM). After 6 h or 12 h, cells were incubated with 10 µM H_2_DCFDA for 30 min at 37 °C in a CO_2_ incubator. Cells were washed three times with PBS to remove unbound probes and digested with trypsin. The intensity of dichlorodihydrofluorescein (DCF) fluorescence was detected by CytoFLEX flow cytometry (Beckman Coulter).

### Statistical analysis

All experiments were repeated three times, and the data are expressed as the mean ± standard deviation (SD). All statistical graphs were generated using GraphPad Prism 7 software (La Jolla, CA, USA). Student’s *t*-test was used for analysis of the significant difference between the groups, and one-way analysis of variance (ANOVA) was performed for comparisons in multiple groups. *P* < 0.05 was considered statistically significant.

## Results

### Her exhibits cytotoxicity to PDAC cells

**Fig. 1:**
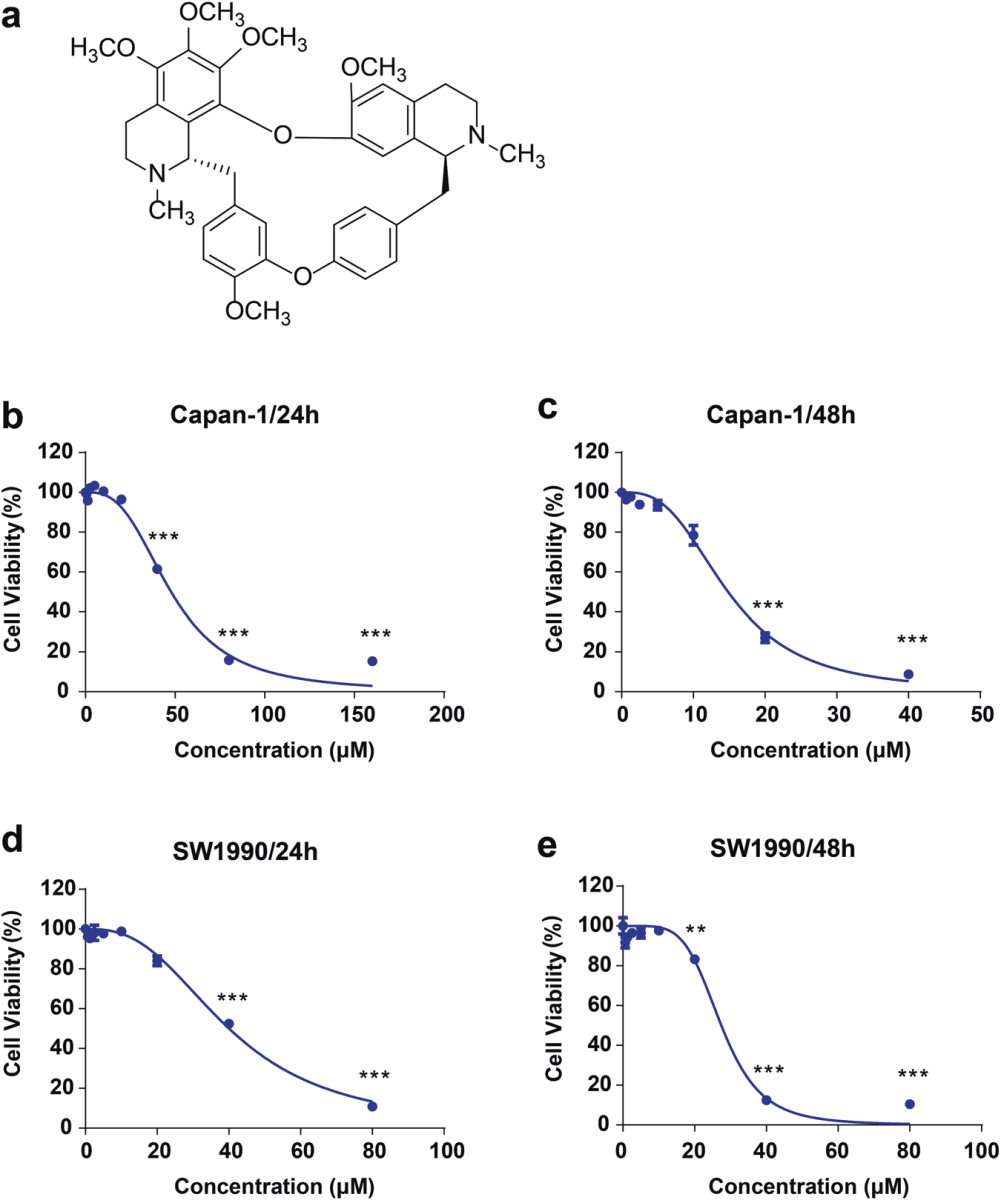
Her inhibits the proliferation of PDAC cells. **a** Chemical structure of Her. **b**, **c** Capan-1 and **d**, **e** SW1990 cells were treated with various concentrations of Her for 24 h and 48 h, respectively. Cell viability was detected by a CCK-8 assay. Data are presented as the mean ± SD, *n* = 3. **P < 0.01, ***P < 0.001 vs. the control group.

Her is a type of bisbenzylisoquinoline alkaloid. The molecular formula of Her is C_39_H_44_N_2_O_7_ and the molecular weight is 652.79 Da. The structural formula of Her is shown in Fig. 1a.

To investigate the effect of Her on the proliferation of Capan-1 and SW1990 cells, two PDAC cell lines, we performed CCK-8 assays. Treatment of Capan-1 and SW1990 cells with Her for 24 h inhibited the proliferation of cells in a dose-dependent manner (Fig. 1b, d). The half-maximal inhibitory concentrations (IC_50_) in Capan-1 and SW1990 cells were 47.7 μM and 40.1 μM, respectively. The proliferation inhibitory effect was enhanced after 48 h of Her treatment (Fig. 1c, e), and the corresponding IC_50_ values in Capan-1 and SW1990 cells were 14.8 μM and 27.5 μM, respectively. These data indicated that Her had strong cytotoxicity to PDAC cells.

### Her induces autophagy in PDAC cells

**Fig. 2:**
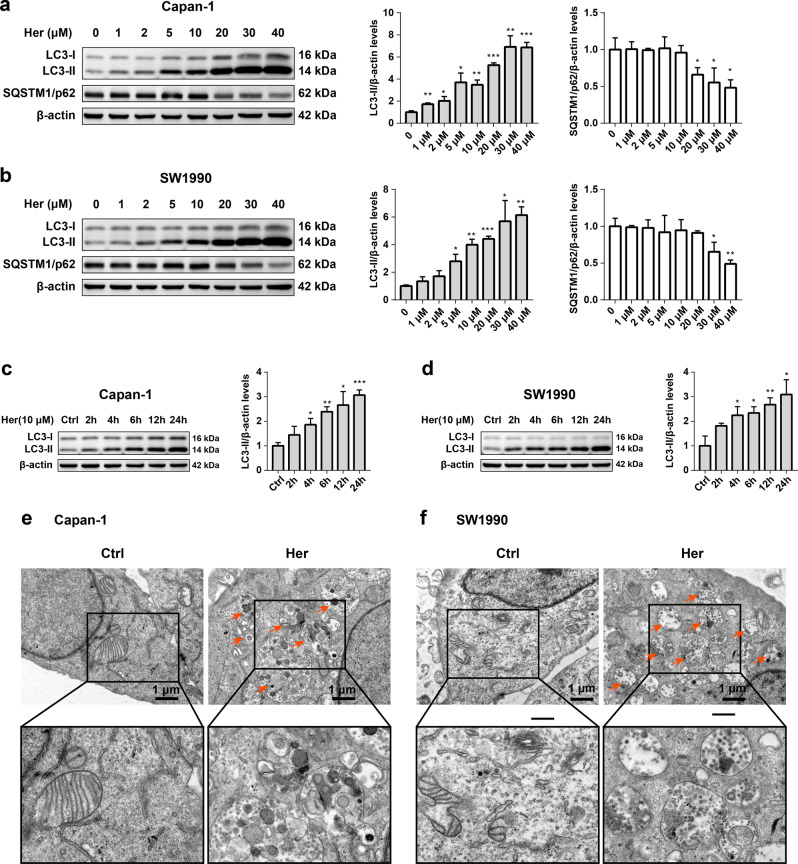
Her induces autophagy in PDAC cells. **a**, **b** Capan-1 and SW1990 cells were treated with various concentrations of Her (0–40 μM) for 24 h. Western blot analysis of LC3-I, LC3-II and SQSTM1/p62 levels in Capan-1 and SW1990 cells. **c**, **d** Capan-1 and SW1990 cells were treated with Her (10 μM) for 2 h, 4 h, 6 h, 12 h and 24 h, and Western blot analysis of LC3-I and LC3-II levels was performed. **e**, **f** Capan-1 and SW1990 cells were treated with Her (10 μM) for 24 h, and autophagic vacuoles (red arrow) in cells were observed by TEM. Data are presented as the mean ± SD, *n* = 3. *P < 0.05, **P < 0.01, ***P < 0.001 vs. the control group.

To examine whether Her triggers autophagy in PDAC cells, we performed Western blot to evaluate the conversion of LC3-I to LC3-II and the level of SQSTM1/p62 in Capan-1 and SW1990 cells treated with Her. Her significantly enhanced the accumulation of LC3-II and decreased the level of SQSTM1/p62 in a dose-dependent manner (Fig. 2a, b). Following previous studies [10] and our results, we selected 10 μM Her for subsequent experiments. We found that LC3-II increased after 24 h of 10 μM Her treatment in PDAC cells (Fig. 2c, d).

To further examine the activation of autophagy induced by Her, we examined autophagosomes and autophagic lysosomes using TEM [35]. Few autophagosomal structures were observed in control cells (Fig. 2e, f). However, large numbers of autophagic lysosomes and double-membrane autophagosomes were detected in the Her treatment groups. Together, these data suggested that Her induces autophagy in PDAC cells.

**Fig. 3:**
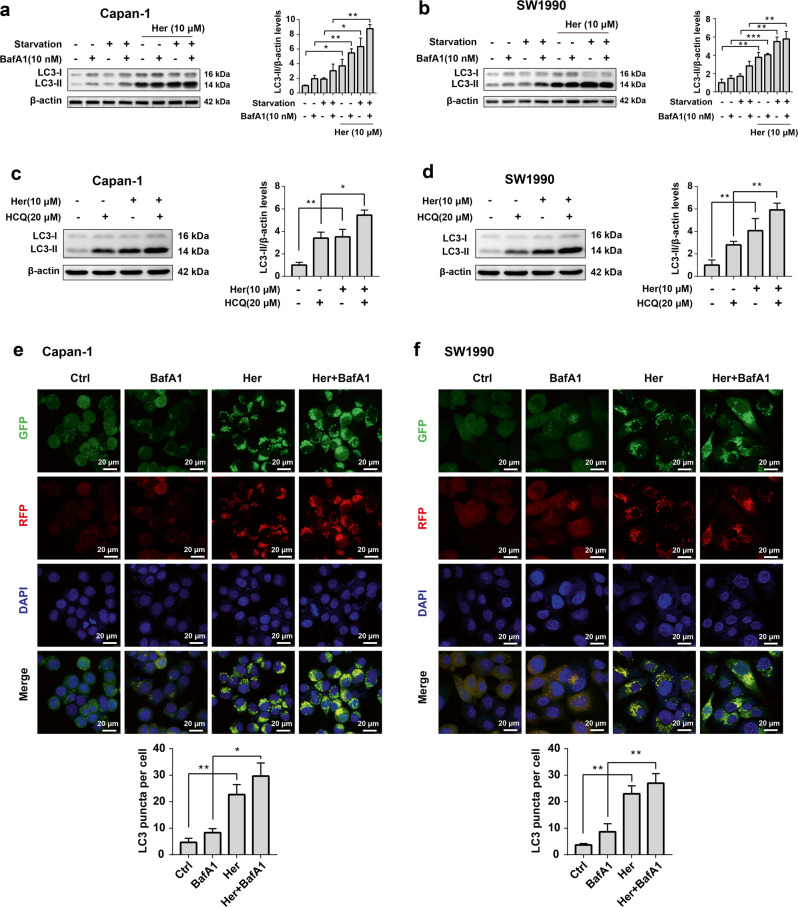
Her in combination with autophagy inhibitors to treat PDAC cells and analyze autophagy activity. **a**, **b** Capan-1 and SW1990 cells were treated with 10 μM Her for 24 h, followed by treatment with 10 nM BafA1 for 3 h. For starvation experiments, cells were incubated in serum-free medium for 1 h, followed by incubation with 10 nM BafA1 for 3 h. **c**, **d** Capan-1 and SW1990 cells were incubated with 10 μM Her for 12 h, followed by incubation with 20 μM HCQ for 12 h. Western blot analysis of LC3-I and LC3-II levels in cells. **e**, **f** GFP-RFP-LC3 puncta were observed using a laser scanning confocal microscope. Data are presented as the mean ± SD, *n* = 3. *P < 0.05, **P < 0.01, ***P < 0.001 vs. the control group.

To further clarify that Her activates autophagy, we used two autophagy inhibitors. BafA1 and HCQ are late-stage autophagy inhibitors that inhibit the autophagy-lysosome pathway and suppress the degradation of LC3-II. BafA1 and HCQ markedly induced the accumulation of LC3-II in Capan-1 and SW1990 cells after treatment with Her (Fig. 3a–d). Starvation is a well-established condition that triggers autophagy [35, 36]. Serum starvation medium–induced autophagy was enhanced in PDAC cells when combined with Her treatment (Fig. 3a, b).

The GFP-RFP-LC3 lentivirus allows for visualization of autophagic puncta under autophagic conditions. Confocal laser microscopy revealed that Her promoted the accumulation of GFP-RFP-LC3 autophagic puncta in Capan-1 and SW1990 cells (Fig. 3e, f). The number of autophagic puncta was further increased in the presence of BafA1. These results suggested that Her strongly induces autophagy in PDAC cells.

### Downregulation of ATG5 relieves autophagy and cell death induced by Her in PDAC cells

**Fig. 4:**
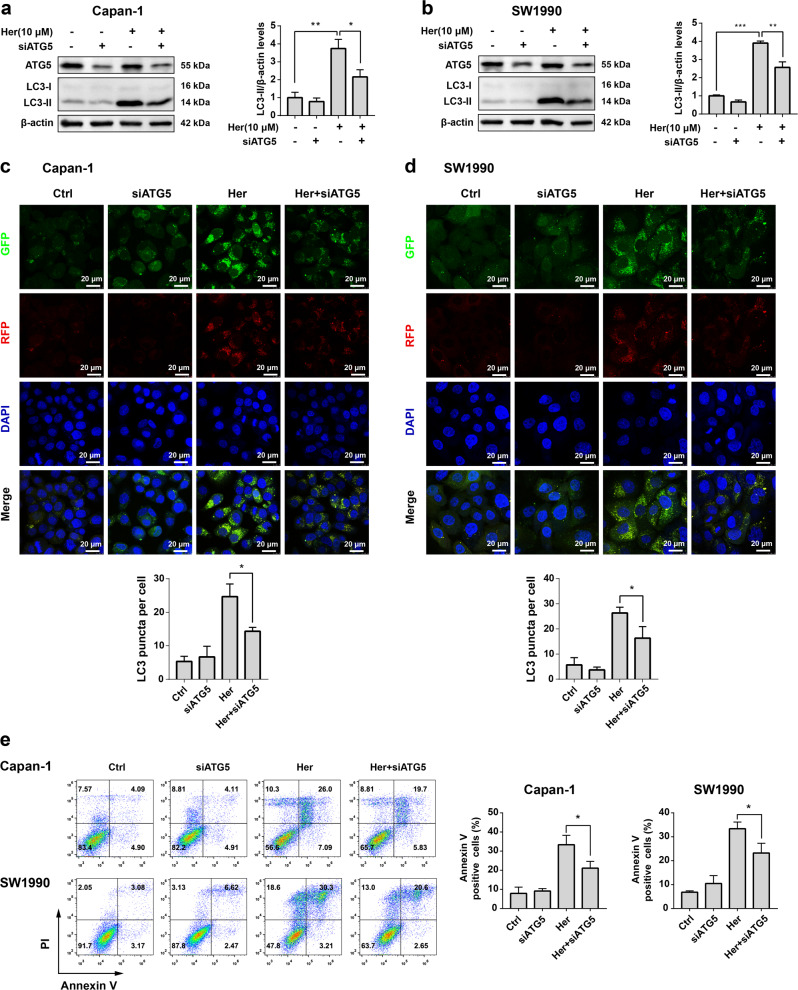
Knockdown of ATG5 relieves autophagy and cell death induced by Her in PDAC cells. Capan-1 and SW1990 cells were transfected with 150 nM siATG5 for 24 h, followed by treatment with 10 μM Her for 24 h. **a**, **b** Western blot analysis of ATG5, LC3-I and LC3-II levels in Capan-1 and SW1990 cells. **c**, **d** GFP-RFP-LC3 puncta were observed using a laser scanning confocal microscope. **e** Capan-1 and SW1990 cells were transfected with 150 nM siATG5 for 24 h and then treated with 25 μM Her and 40 μM Her for 24 h, respectively. Cell death was measured with Annexin V-FITC and propidium iodide (PI) staining and flow cytometry. Data are presented as the mean ± SD, *n* = 3. *P < 0.05, **P < 0.01, ***P < 0.001 vs. the corresponding group.

ATG5 has been shown to play an important role in autophagy. The lack of ATG5 causes cancer cells to be insensitive to drug-induced autophagy [37]. To examine the role of ATG5 in Her-induced autophagy, we knocked down expression of the ATG5 gene using siRNA in PDAC cells. The expression level of ATG5 in both Capan-1 and SW1990 cells was decreased by siATG5 (Fig. 4a, b). Downregulation of ATG5 inhibited Her-induced autophagy in PDAC cells, as indicated by the alleviation of the Her-induced increase in LC3-II expression by ATG5 knockdown (Fig. 4a, b). In addition, siATG5 inhibited the formation of GFP-RFP-LC3 autophagic puncta induced by Her in Capan-1 and SW1990 cells (Fig. 4c, d). Moreover, downregulation of ATG5 alleviated Her-induced cell death to a certain extent (Fig. 4e). These data further indicated that Her induced autophagy and autophagic cell death, and down-regulation of ATG5 expression inhibited the activation of autophagy by Her.

### Autophagy inhibitors decreased the cell death induced by Her in PDAC cells

**Fig. 5:**
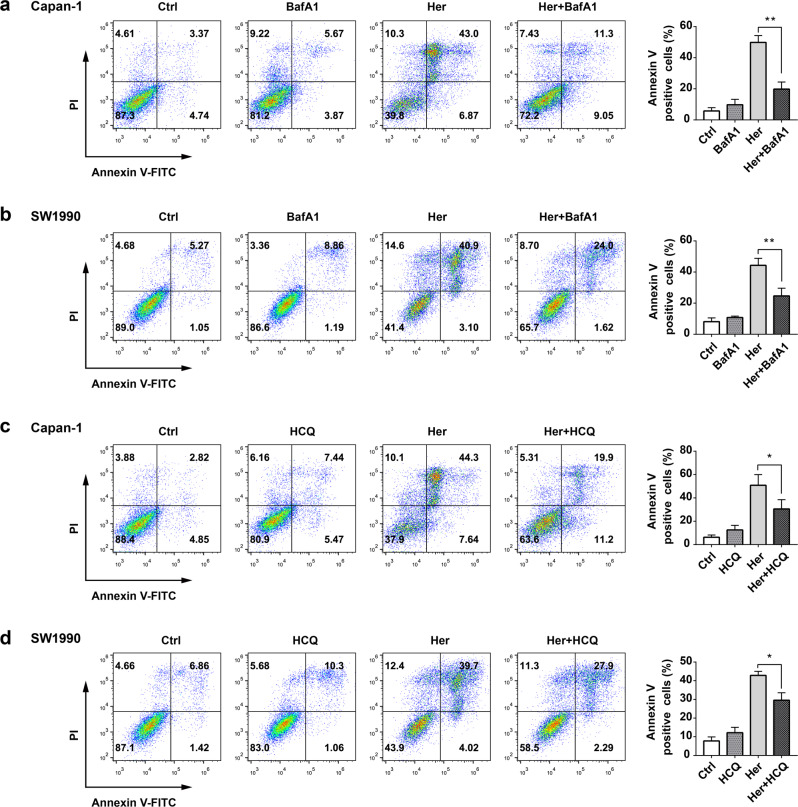
Autophagy inhibitors decreased the autophagic cell death induced by Her in PDAC cells. **a**, **c** Capan-1 cells were treated with 30 μM Her in the presence or absence of 10 nM BafA1 or 100 μM HCQ for 24 h. **b**, **d** SW1990 cells were treated with 50 μM Her in the presence or absence of 10 nM BafA1 or 100 μM HCQ for 24 h. Flow cytometry was performed to analyze cell death with Annexin V-FITC and PI staining. Data are presented as the mean ± SD, *n* = 3. *P < 0.05, **P < 0.01 vs. the corresponding group.

Capan-1 and SW1990 cells were incubated with 30 μM and 50 μM Her for 24 h, respectively, and resulted in close to half of the Annexin V-positive rate (Fig. 5a–d). BafA1 is a late-stage inhibitor of autophagy that blocks the fusion of autophagosomes into lysosomes and inhibits both acidification and protein degradation in lysosomes. HCQ is also an inhibitor of autophagy at the late stage that suppresses the autophagic process by preventing lysosomal acidification. Both BafA1 and HCQ promote cell apoptosis. Our results showed that BafA1 (10 nM) decreased the cell death induction by Her in Capan-1 and SW1990 cells (Fig. 5a, b) and HCQ (100 μM) also reduced the cell death induced by Her (Fig. 5c, d). These results show that the autophagy inhibitors BafA1 and HCQ significantly decreased the induction of cell death by Her, indicating that Her induced autophagic cell death in PDAC cells.

### Her activates the AMPK signaling pathway and inhibits the mTOR signaling pathway in PDAC

**Fig. 6:**
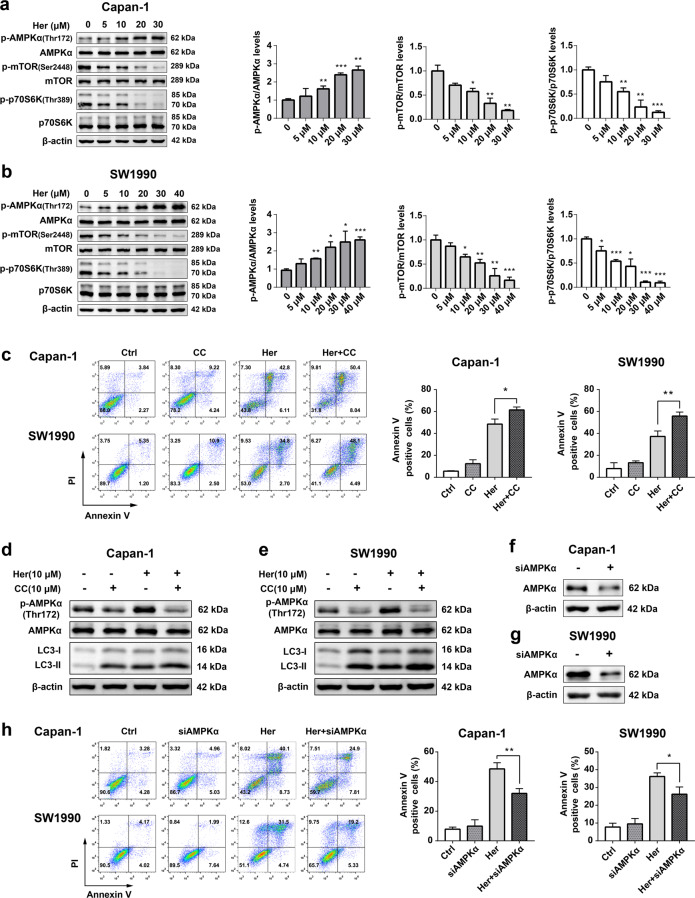
Her triggered autophagy and autophagic cell death by activating the AMPK/mTOR signaling pathway. **a**, **b** Capan-1 and SW1990 cells were treated with various concentrations of Her (0–40 μM) for 12 h. Western blot analysis of p-AMPKα (Thr172), AMPKα, p-mTOR (Ser2448), mTOR, p-p70S6K (Thr389) and p70S6K levels. **c** Capan-1 and SW1990 cells were treated with 30 μM Her and 40 μM Her in the presence or absence of 10 μM Compound C for 24 h, respectively. Flow cytometry was performed to measure cell death with Annexin V-FITC and PI staining. **d**, **e** Capan-1 and SW1990 cells were treated with 10 μM Her in the presence or absence of 10 μM Compound C for 24 h. Western blot analysis of p-AMPKα (Thr172), AMPKα, LC3-I and LC3-II levels. **f**, **g** Capan-1 and SW1990 cells were transfected with 150 nM siAMPKα for 24 h. Western blot analysis of AMPKα in Capan-1 and SW1990 cells. **h** Capan-1 and SW1990 cells transfected with 150 nM siAMPKα were treated with 30 μM Her and 40 μM Her for 24 h, respectively. Flow cytometry was performed to measure cell death. Data are presented as the mean ± SD, *n* = 3. *P < 0.05, **P < 0.01, ***P < 0.001 vs. the corresponding group.

To determine whether Her-induced autophagy involves the AMPK/mTOR signaling pathway, we evaluated levels of phospho-AMPKα (p-AMPKα), phospho-mTOR (p-mTOR) and phospho-p70S6K (p-p70S6K) by Western blot. As the concentration of Her increased, the level of p-AMPKα was enhanced and the levels of p-mTOR and p-p70S6K decreased in Capan-1 and SW1990 cells (Fig. 6a, b). These results suggested that Her activated the AMPK signaling pathway and inhibited the mTOR/p70S6K signaling pathway.

### CC enhances autophagy and cell death induced by Her in PDAC cells

To identify the role of AMPK in Her-induced autophagy and cell death in PDAC cells, we used the AMPK inhibitor CC for further investigation. Capan-1 cells were incubated with 30 μM Her in the presence or absence of 10 μM CC for 24 h. The results showed that CC did not inhibit Her-induced cell death but instead increased Her-induced cell death in Capan-1 cells (Fig. 6c). The results in SW1990 cells were similar to the results in Capan-1 cells.

CC inhibits AMPK phosphorylation but also activates autophagy in cells. Capan-1 and SW1990 cells were incubated with 10 μM Her and 10 μM CC for 24 h. Western blot analysis revealed that CC decreased the phosphorylation level of AMPK induced by Her (Fig. 6d, e). CC also promoted the conversion of LC3-I to LC3-II and enhanced autophagy. These results showed that both CC and Her induced autophagy and CC promoted Her-induced autophagic cell death in PDAC cells.

### Knockdown of AMPK decreased Her-induced autophagic cell death in PDAC cells

To further investigate whether AMPK was involved in the effects of Her in PDAC, we used siRNA to downregulate the AMPKα gene in PDAC cells and confirmed that siAMPKα decreased the expression level of AMPKα in both Capan-1 and SW1990 cells (Fig. 6f, g). Furthermore, downregulation of AMPKα decreased the percentage of Annexin V-positive cells induced by Her (Fig. 6h). This result implied that knockdown of AMPK caused PDAC cells to be insensitive to autophagic cell death induced by Her.

### Her promotes ROS accumulation in PDAC cells

**Fig. 7:**
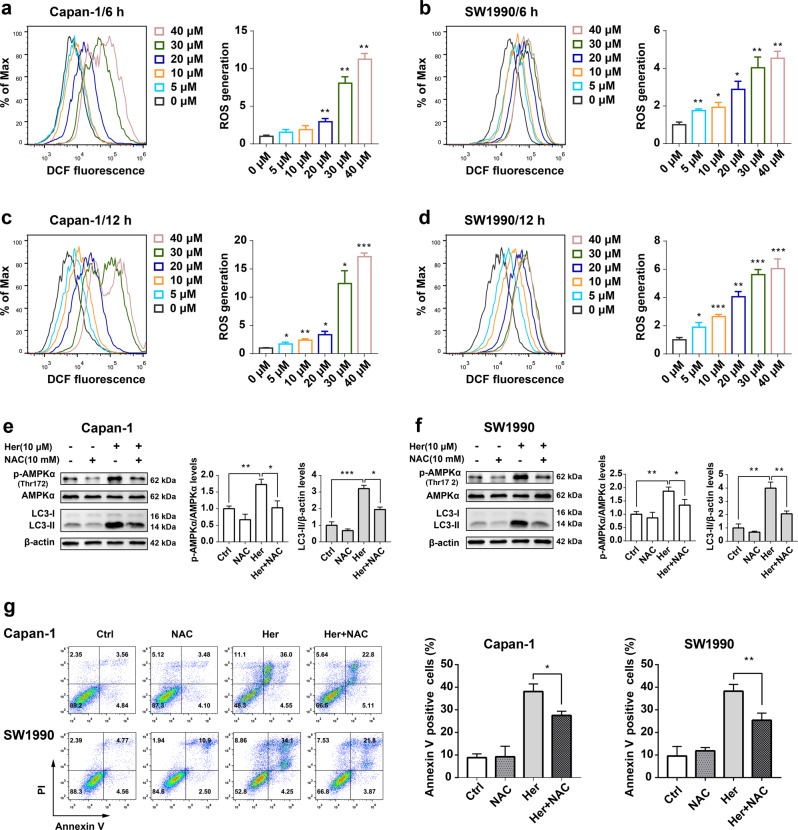
Her generated ROS and NAC treatment decreased autophagy in PDAC cells. **a**, **c** Capan-1 and **b**, **d** SW1990 cells were treated with various concentrations of Her (0, 5, 10, 20, 30, 40 μM) for 6 h and 12 h. ROS production in Capan-1 and SW1990 cells was detected by flow cytometry to detect DCF fluorescence intensity. **e**, **f** Capan-1 and SW1990 cells were treated with 10 μM Her in the presence or absence of 10 mM NAC for 24 h. Western blot analysis of p-AMPKα (Thr172), AMPKα, LC3-I and LC3-II levels in Capan-1 and SW1990 cells. **g** Capan-1 and SW1990 cells were treated with 25 μM Her and 40 μM Her in the presence or absence of 10 μM NAC for 36 h, respectively. Flow cytometry was performed to measure cell death with Annexin V-FITC and PI staining. Data are presented as the mean ± SD, *n* = 3. *P < 0.05, **P < 0.01, ***P < 0.001 vs. the corresponding group.

To investigate whether Her led to ROS accumulation in Capan-1 and SW1990 cells, we used the H_2_DCFDA probe, which can freely enter cells and be oxidized by intracellular ROS to DCF. The fluorescence intensity of DCF detected by flow cytometry reflects the level of ROS in cells. After Her was incubated with cells for 6 h and 12 h, ROS production increased in Capan-1 and SW1990 cells in a concentration-dependent manner (Fig. 7a–d). These data suggested that Her-induced ROS accumulation in PDAC cells.

### NAC relieves the AMPK phosphorylation and autophagy induced by Her in PDAC cells

**Fig. 8:**
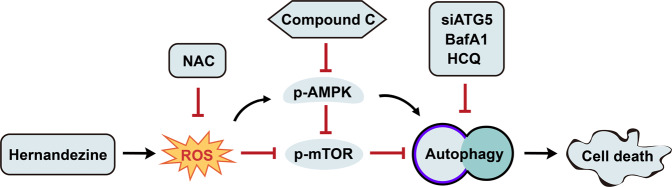
Diagram of the proposed mechanism by which Her induces autophagic cell death in PDAC cells. Her treatment results in ROS accumulation, which regulates the AMPK/mTOR pathway and induces autophagy in PDAC cells. NAC, siATG5, BafA1 and HCQ inhibit autophagy induced by Her.

Our results showed that Her induced autophagy, activated the AMPK signaling pathway and significantly enhanced ROS generation in Capan-1 and SW1990 cells. We further investigated the association between ROS and autophagy. The antioxidant NAC can scavenge ROS generated in cells. Treatment of cells with 10 μM Her for 24 h increased the phosphorylation level of AMPKα in Capan-1 and SW1990 cells; when cells were co-treated with 10 mM NAC, the expression levels of p-AMPK and LC3-II decreased in cells (Fig. 7e, f). Moreover, while Her induced autophagic cell death, NAC attenuated this effect (Fig. 7g). These data revealed that NAC, an ROS scavenger, suppressed autophagy induced by Her in PDAC cells. These results suggested that ROS might be an upstream signaling molecule in Her-induced autophagy in PDAC cells (Fig. 8).

## Discussion

Her is a bisbenzylisoquinoline alkaloid extracted from traditional Chinese herbal medicine. Her has been reported to have immunosuppressive effects [38], inhibit platelet aggregation [11] and reverse tumor multidrug resistance [10, 14, 39]. However, the effects of Her on pancreatic cancer have not yet been reported. This study revealed that Her inhibited cell proliferation and induced autophagic cell death in PDAC cells. In subsequent studies, we will focus on the application of Her alone or in combination with clinically available drugs for pancreatic cancer treatment.

Previous studies showed that Her is a potent activator of AMPK [5, 10]. Our study also demonstrated that Her activates AMPK phosphorylation in PDAC cells. Increased AMPK phosphorylation induces apoptosis and promotes autophagy [37, 40]. Our data showed that Her markedly activated autophagy in PDAC cells, and Her-mediated activation of autophagy was time-dependent and dose-dependent. LC3 and SQSTM1/p62 are two marker proteins for the assessment of autophagy activity [41, 42]. We detected the conversion of LC3-I to LC3-II in cells treated with 10 μM Her for 24 h. However, SQSTM1/p62 was not significantly degraded until cells were treated with Her above 20 μM, which may be the result of Her promoting the expression of SQSTM1/p62 so that the content of SQSTM1/p62 is still high during the process of autophagy. In our next studies, we plan to analyze the effect of Her on SQSTM1/p62 expression through RNA sequencing. Western blot analysis of PDAC cells treated with Her combined with late-stage autophagy inhibitors BafA1 and HCQ revealed that Her promoted the conversion of LC3-I to LC3-II. In addition, Her increased the formation of GFP-RFP-LC3 puncta as detected by confocal microscopy. TEM observation showed that autophagosomes and autophagic lysosomes were dramatically increased in PDAC cells after Her treatment. ATG5 is a key factor in the autophagy signaling pathway [43]. We found that the autophagy activated by Her was attenuated in PDAC cells by knockdown of ATG5 by siRNA. These results indicated that Her induces autophagy in PDAC cells.

Research has shown that autophagy has a dual effect on cells. Autophagy plays a cytoprotective role, but it may also lead to cytotoxicity [44]. Our results suggest that Her strongly activates autophagy in PDAC cells. To determine whether Her-induced autophagy leads to autophagic cell death, cells were treated with Her in combination with the autophagy inhibitors BafA1 and HCQ. The results showed that either BafA1 or HCQ inhibited Her-induced cell death. This result indicated that Her induced autophagic cell death in PDAC cells. Western blot analysis showed that Her increased the phosphorylation of AMPK and decreased the phosphorylation of mTOR and p70S6K, thereby activating autophagy in PDAC cells. These findings are consistent with those of existing studies [10]. When PDAC cells were treated with the AMPK inhibitor CC, CC promoted Her-induced cell death, which is inconsistent with the results reported by Law et al.; however, the cancer cells used in the previous study were different from the cancer cells in the present study, and the cells they used are drug-resistant [10]. CC has been reported to promote autophagy [45, 46]. Our Western blot analysis showed that CC promoted autophagy in PDAC cells, and CC enhanced the induction of autophagy caused by Her. Therefore, we speculate that although CC inhibits AMPK phosphorylation, CC cooperates with Her to induce autophagy and promote autophagic death in PDAC cells. When AMPK expression was downregulated using RNAi, PDAC cells were insensitive to Her-induced death.

Her analogs enhance ROS generation in cells [29–31]. We speculated that there are regulatory factors between Her and AMPK phosphorylation, and ROS may be one of the key factors. ROS participate in the regulation of autophagy through multiple signaling pathways, including the AMPK and mTOR/p70S6K pathways [33, 47, 48]. Therefore, we examined ROS production in PDAC cells after Her treatment with the H_2_DCFDA probe, and the results showed that ROS production in cells was positively correlated with the concentration of Her. After treatment of NAC (an ROS scavenger) with Her, the phosphorylation level of AMPK in cells was reduced, the conversion of LC3-I to LC3-II was decreased and the percentage of Annexin V-positive cells was also decreased. These results suggest that Her induces autophagy and autophagic cell death by promoting the production of ROS in PDAC cells.

This study revealed that Her activates autophagy and induces autophagic cell death in PDAC cells by promoting ROS generation, activating the AMPK signaling pathway and inhibiting the mTOR/p70S6K signaling pathway. Autophagy inhibitors can significantly alleviate Her-induced autophagic cell death in PDAC cells. The present study provides powerful evidence for the development of Her as a therapeutic agent for pancreatic cancer.
